# Advancing AAV technology: From capsid design to scalable manufacturing

**DOI:** 10.1016/j.omtm.2025.101477

**Published:** 2025-05-07

**Authors:** Daniel Stone, Mario Mietzsch, Giuseppe Ronzitti

**Affiliations:** 1 Vaccine and Infectious Disease Division, Fred Hutchinson Cancer Center, Seattle, WA, USA; 2 Department of Biochemistry and Molecular Biology, University of Florida, Gainesville, FL, USA; 3 Genethon, 91000 Evry, France; 4 Université Paris-Saclay, University Evry, Inserm, Genethon, Integrare Research Unit UMR_S951, 91000 Evry, France

Since its launch as the second sibling journal to *Molecular Therapy* in 2014 over 10 years ago,^1^ *Molecular Therapy—Methods and Clinical Development* (*MTMCD*) has served as a platform for the reporting of high-quality scientific developments by the gene and cell therapy community. Under the stewardship of its founding editor-in-chief Matt Porteus, then subsequent editors-in-chief Roland Herzog and Gerhard Bauer, and now Mohamed Abou-el-Enein, *MTMCD* has established itself as an important member of the *Molecular Therapy* family of journals. While much of the content of *MTMCD* is methods and technology focused, our overall scope is broad, encompassing all areas of basic, translational, and clinical research related to gene and cell therapies. This enables us to publish important papers across many different categories within our field.

This first of two adeno-associated virus (AAV)-focused special collections highlights papers published in *MTMCD* since 2024 featuring advances in capsid structure, engineering, production, and large-scale manufacturing of therapeutic AAV vectors. In the continually evolving world of gene and cell therapy, AAV vectors are undoubtedly the most widely used gene delivery system, and *MTMCD* is proud to be a platform for the publication of many papers that demonstrate developments in the use of AAV as a therapeutic agent. This first of two collections focused on AAV has been put together by renowned AAV experts from our scientific editorial team, who have summarized some of their favorite AAV advances featured in this collection.

*AAV structure*: AAV vectors are derived from wild-type adeno-associated viruses, except that the viral single-stranded DNA genome is replaced with a therapeutic expression vector cassette for the treatment of genetic diseases. The vector genome is enclosed within an icosahedral protein shell, called the capsid. Within the capsid the packaged genome is protected from nucleases and other environmental factors. Furthermore, the exact amino acid composition of the capsid determines the mode of purification, its ability to bind specific receptors on the surface of target cells, and ultimately which tissue or organs can be treated. The 3D-structures of several AAV capsids have been determined. However, as multifunctional proteins, the capsid structures require annotation to pinpoint the important regions affecting the steps from AAV vector production to administration. This special *MTMCD* collection contains several studies further characterizing the capsids of the AAVs. Yamaguchi and colleagues studied the glycosylation of AAV6 capsids, post-translational modifications whose impact on capsid function remains incompletely understood.^2^ In their study, they found a comparable glycosylation profile in six independent AAV6 vector preparations and identified specific amino acids with attached O-glycans. While the percentage of capsids with these modifications is low, they were able to separate these capsids, with or without mucin-type O-glycans. Yamaguchi and colleagues showed that capsids with the attached glycans displayed reduced transduction efficiency *in vitro* and *in vivo*, indicating that the glycosylation of AAV vector may require further attention for effective gene transfer.

AAV capsids are a target of numerous engineering efforts to improve the ability of the vectors to deliver their therapeutic gene to target cells. Toward these efforts, La Bella and colleagues analyzed two libraries of natural AAV2 and AAV13 capsid variants.^3^ Characterizing these variants provides insight into which capsid regions are amenable to modification. In this study, the authors developed a prediction method to estimate the fitness of AAV2 capsids containing multiple amino acid changes. This tool may be useful for the development of AAV capsid libraries to enrich for viable variants, thus accelerating the design of engineered capsids.

The introduction of amino acid changes to the capsid may also affect the purification strategy for a selected AAV vector. To further guide these engineering efforts, Mietzsch and colleagues determined the binding site of the AAVX affinity ligand and the capsid of AAV8 by cryoelectron microscopy.^4^ The ligand binds near the 5-fold axis of the capsid, primarily interacting with the peptide backbone of the conserved DE- and HI-loops, thus explaining the cross-reactivity to multiple AAV serotypes. Affinity chromatography based on the AAVX ligand is one of the most utilized methods for AAV vector purification. The identification of the binding epitope may guide the capsid selection process by excluding potential capsids incapable of being purified with AAVX.

*AAV capsid engineering*: The broad cell tropism of AAV vectors has long been one of the features that made them a preferred choice for *in vivo* gene therapy. However, as clinical and preclinical experience continues to grow, it has become evident that improving cell-specific targeting—while narrowing non-specific distribution—is essential for achieving safe and effective gene transfer across the broader patient population. To this end, multiple approaches have been developed to identify novel or engineered capsids with desired tropisms via library-based screening in the target cell type or tissue of choice.^5^^,^^6^

One notable example of ongoing efforts to enhance AAV vectors is the recent identification of myotropic capsids. A growing list of capsids with improved muscle-targeting capabilities—and, in some cases, reduced liver off-targeting—is now available to researchers in the neuromuscular field. Advances in characterizing muscle-specific targeting are exemplified by studies like the one by McGowan and colleagues,^7^ in which biodistribution was evaluated at the cellular level by isolating muscle-resident mononuclear cells. The level of detail shown in their analysis is especially important for therapeutic applications, as off-targeting of vector to undesired cell populations may negatively influence clinical outcomes. While transgene expression in immune and endothelial cells can affect the immune response following AAV treatment, the specific targeting of muscle stem cells seen is particularly relevant for gene editing in neuromuscular diseases, as it provides a corrected cell reservoir capable of regenerating diseased muscle tissue.

Another example of an approach to improve AAV vector specificity involves the use of anti-fibroblast activation protein (FAP) nanobodies to selectively target tumors.^8^ Although AAV vectors are not commonly used in cancer therapy, advances in vector engineering could enhance their ability to target tumors by enabling the targeted delivery of suicide genes or immune-modulating agents directly to cancer cells. In rodent models, modified AAV2 vectors targeting the tumor microenvironment via FAP —specifically stromal fibroblasts—showed promise, offering a potential new tool in the expanding arsenal of anti-cancer strategies. This study also underscores how AAV vectors can be customized to efficiently target specific cell types while minimizing off-target effects.

*AAV vector production*: Whether being produced at small scale in a laboratory, or at large scale in a cGMP facility, AAV can be manufactured via several different platforms, with the most commonly used platforms requiring triple plasmid transfection of HEK293 cells, baculovirus infection of Sf9 cells, or herpes simplex virus helper virus infection of producer cells. Although AAV production using these platforms is well established, the inherent inefficiency of AAV infection requires high virus-to-cell ratios, often necessitating the generation of large quantities of therapeutic vector. Thus, researchers are constantly developing methods to make the production of AAV both more cost effective and more efficient. Several examples of methods to improve AAV vector production are included in this special *MTMCD* collection. In a study by Liu et al., an optimized protocol is presented that not only allows for the production of AAV-inhibitory transgenes but also has the potential to significantly reduce the costs associated with production of AAV via the triple plasmid transfection method in HEK293 producer cells.^9^ They demonstrate that after transfection of HEK293 cells, the input AAV vector DNA plasmid is replicated during the production phase, enabling the use of significantly less vector DNA plasmid than normal. When the amount of vector DNA plasmid used during transfection is reduced by 10- to –100-fold, comparable AAV yields are seen, with less unwanted packaging of plasmid backbone DNA into virions. In a study by Kuz and colleagues, different improvements related to vector production were described. Two host soluble N-ethylmaleimide-sensitive factor attachment protein receptors (SNARE) proteins were identified, STX7 and SNAP23, which indirectly interact with the AAV2 membrane-associated accessory protein (MAAP) protein that facilitates virus egress from the cell through an association with extracellular vesicles.^10^ The authors showed that AAV2 MAAP interacts with STX7 and SNAP23 inside the cytoplasm at the plasma membrane and when the authors knocked out STX7 or SNAP23 expression from AAV producer cells, they saw an increase in vector yields as well as an increase in the levels of vector secreted into the medium for several different AAV serotypes. These are just two examples of the methodological advances in AAV production that are included in this special collection of *MTMCD* papers.

*AAV vector purification and quality control*: The purification and subsequent validation of therapeutic AAV vectors are essential steps in the production of research and clinical grade AAV-based therapies. Whether producing AAV for small scale animal studies or for large-scale clinical use, AAV that is being administered as a therapeutic should be highly pure and well characterized both physically and biologically. Examples of new methods to improve AAV vector purification and quality control are included in this special *MTMCD* collection.

Toward the improvement of AAV vector purification, a study by Thakur and colleagues developed a strategy to enrich the ratio of full capsids.^11^ Empty capsids are a common byproduct of AAV vector productions that lack the enclosed vector genome. These capsids do not provide any curative benefit and may even elicit unwanted immune reactions. Thakur and colleagues demonstrate the utilization of a two-pass anion exchange chromatography method for AAV1, AAV2, AAV8, and AAV9 to enrich AAV preparations from low to over 90% full capsids. The authors show the applicability of this method for up to 500 L productions to obtain vector titers in the e15–e16 vg/L range, suitable for the rapid generation of preclinical, clinical, and commercial material for therapeutic AAVs.

Efforts to fully characterize contaminants in AAV vector preparations continue, as demonstrated in two studies highlighted in this collection. The first study by Leibiger and colleagues^12^ presents an in-depth analysis of host-cell protein contaminants remaining after affinity chromatography of AAV produced in HEK293 cells. Notably, many of the most abundant proteins identified were associated with the heat shock response, suggesting either a direct interaction with the AAV capsid or a stress-induced response in the producer cell line during vector production. The second study, by Penaud-Budloo and colleagues,^13^ reports the intriguing discovery of microRNA (miRNA) contaminants in AAV vector preparations produced in both HEK293 cells and Sf9 cells via the baculovirus system. Although the direct impact of these miRNAs on the safety of AAV gene therapy may be limited, their transient expression in target cells could alter the cells’ transcriptome and potentially mark them for elimination by the innate immune system. This process may contribute to early immune responses against the vector, which in turn could trigger a more robust adaptive immune reaction—ultimately affecting both the safety and efficacy of AAV-mediated gene transfer.

In a study by Dunker-Seidler and colleagues, a method is described for long-read nanopore sequencing of AAV vector genomes within AAV preps that uses new V14 sequencing chemistry and the dorado base calling algorithm.^14^ When compared with older V9 chemistry, V14-based nanopore sequencing had higher raw read quality, which enhanced the average alignment match rate from 96.8% to 98.0% and reduced the average rate of indels from 1.42% to 0.55%. The authors compared their modified nanopore sequencing protocol to the gold standard long-read single molecule real-time (SMRT) approach and found that, despite its lower raw read quality, it provided a comparable overview of within-batch AAV genome content, particularly for identifying encapsidated DNA impurities. When the overall costs of different long-read sequencing approaches are considered, this modified nanopore approach may offer a significantly lower priced alternative to the current gold standard.

We hope this first AAV-focused collection offers valuable insights into the engineering, production, and manufacturing of AAV vectors for investigators working to advance the field. The upcoming second collection will spotlight the therapeutic application of AAV, with studies addressing safety and efficacy across preclinical models and clinical settings. As gene and cell therapy continue to mature, *MTMCD* remains at the forefront, publishing pioneering work in AAV vector science. Together, these two collections reinforce *MTMCD*’s position as a leading journal for cutting-edge research driving the next generation of AAV-based therapies.
